# Beyond the Gastrointestinal Tract: The Emerging and Diverse Tissue Tropisms of Astroviruses

**DOI:** 10.3390/v13050732

**Published:** 2021-04-22

**Authors:** Andrew B. Janowski

**Affiliations:** Department of Pediatrics, Washington University School of Medicine, St Louis, MO 63110, USA; abjanowski@wustl.edu; Tel.: +1-314-454-6050

**Keywords:** astroviruses, tropism, gastroenteritis, encephalitis, hepatitis, respiratory disease

## Abstract

Astroviruses are single stranded, positive-sense RNA viruses that have been historically associated with diseases of the gastrointestinal tract of vertebrates, including humans. However, there is now a multitude of evidence demonstrating the capacity of these viruses to cause extraintestinal diseases. The most striking causal relationship is neurological diseases in humans, cattle, pigs, and other mammals, caused by astrovirus infection. Astroviruses have also been associated with disseminated infections, localized disease of the liver or kidneys, and there is increasing evidence suggesting a potential tropism to the respiratory tract. This review will discuss the current understanding of the tissue tropisms for astroviruses and their emerging capacity to cause disease in multiple organ systems.

## 1. Introduction

**Figure 1:**
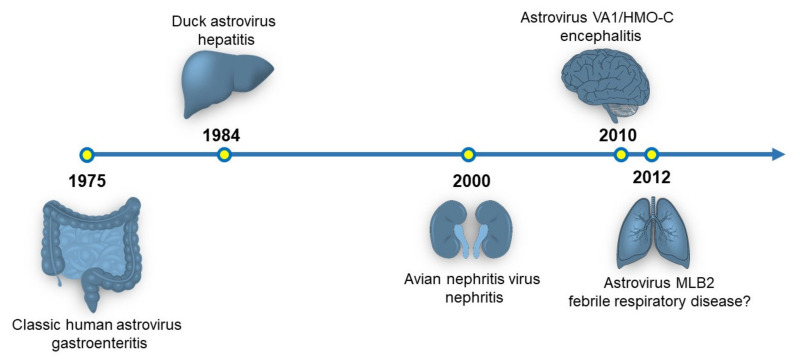
Timeline of the organ systems associated with astrovirus disease. The originally identified astrovirus strain and disease association are shown. Currently, there is a hypothesized link between astrovirus infection and respiratory disease, but no experimental data exists supporting this association.

Since their discovery in 1975, astroviruses have been primarily isolated from the gastrointestinal tract of birds and mammals, including humans [1]. Much of the biology of astroviruses has been described in the context of gastrointestinal infections [2]. However, astroviruses have been identified to cause disease outside of the gastrointestinal (GI) tract and they have been recently recognized to be a novel pathogen of the central nervous system (Figure 1) [2,3]. This review will discuss the currently known tissue tropisms of astroviruses and future directions that may expand the known tropisms for this viral family.

## 2. Astrovirus Genetic Diversity

**Figure 2:**
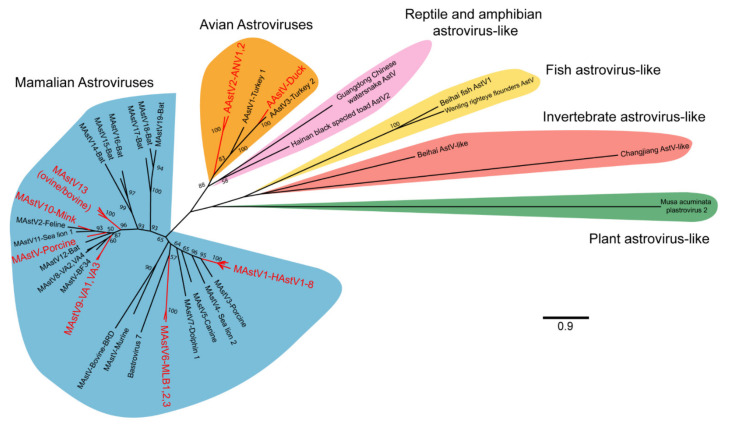
Phylogenetic relationships of representative astrovirus ORF2 sequences. A total of 53 astrovirus genomes were selected for analysis and include the 22 astrovirus species recognized by ICTV, genotypes implicated in extraintestinal diseases, and recently identified representative reptilian, amphibian, fish, invertebrate, and plant astrovirus-like genomes (see Supplementary Materials for full list). Astrovirus amino acid sequences for ORF2 were aligned using MUSCLE and noninformative alignments removed by TrimAI [10,11]. A maximum likelihood tree was generated in MEGA X, using the LG model with frequencies and discrete gamma distribution [12]. A total of 100 bootstraps were completed with bootstrap values >50 represented on the tree. Astrovirus clades that have been implicated in extraintestinal diseases are highlighted in red. MAstV: mamastrovirus; AAstV: avastrovirus; HAstV: human astrovirus; AstV: astrovirus.

The International Committee on the Taxonomy of Viruses (ICTV) currently recognizes two genera of astroviruses, avastrovirus (avian astroviruses) and mamastroviruses (mammalian astroviruses; Figure 2) [4]. However, this naming convention will likely undergo further expansion as newer astrovirus genomes have now been identified from many other vertebrates including reptiles, amphibians, and fish, and from invertebrates (Figure 2) [5,6,7]. In addition, some astrovirus-like viruses have been identified, including those that have evidence of recombination with hepatitis E [8]. Further astrovirus-like genomes have been identified from plants [9]. How these highly divergent viruses will be classified, either as members of the *Astroviridae* family or not remains to be determined. Nonetheless, the genetic diversity of astroviruses is far greater than what was previously known only just a decade ago.

Across the two currently named genera of astroviruses, a total of 22 species are currently recognized (Figure 2) [4]. In general, most astroviruses are named after the host in which they are identified and presumed to infect [1,13]. However, some species of astroviruses can be infrequently identified outside of their primary host, suggesting that astroviruses may have the capacity to infect different host species [13]. For astroviruses that infect humans, they have often been divided between the classic human astroviruses that were first identified in 1975 (human astrovirus 1–8) and the recently discovered novel human astroviruses (VA1-VA4, BF34, MLB1-3). Within the mamastrovirus genus, a clade known as the HMO (human, mink, and ovine) clade has received particular attention given the frequency at which members of this clade are detected from cases of central nervous system infection in humans and other mammals [13]. Members of this clade include some human astroviruses (VA clade), bovine, ovine, mink, and porcine astroviruses that are phylogenetically distinct from other astrovirus strains that infect the same host [13].

## 3. The Role of Astrovirus Biology in Tissue Tropisms

Astroviruses are single-stranded, positive-sense RNA viruses that comprise the family *Astroviridae* [2]. Astrovirus genomes range approximately in size from 6000–7700 nucleotides in length and contain 3–4 open reading frames [2]. ORF1a is a polypeptide that encodes a protease and other non-structural proteins [2]. A slippery sequence between ORF1a and ORF1b allows for ribosomal frameshifting and the subsequent translation of ORF1b, which encodes the RNA-dependent RNA polymerase [2]. ORF2 encodes the structural proteins that comprise the viral capsid [2]. Some astroviruses also contain an additional small open reading frame, ORFX, that encodes a viroporin [14]. During the replication cycle of astroviruses, a subgenomic RNA strand that encompasses ORF2 is transcribed through a highly conserved promotor sequence and is a possible mechanism by which astroviruses upregulate translation of viral capsid proteins [2].

Currently, the genetic determinants that affect the tissue tropisms of astroviruses are unknown from both the perspective of the host and the virus. The host receptor(s) by which astroviruses gain entry to the cell are undefined, so the spectrum of potentially infectable cells based on receptor expression cannot be studied [13]. A small number of host–virus interactions have been described, but the essential factors during the viral lifecycle also remain largely unexplored [2].

Some astroviruses, including the classic human astroviruses, require incubation with trypsin for propagation in cell culture [15]. While this requirement could be an artifact of cell culture, this finding would suggest that there might be host proteases that are essential for completion of the viral lifecycle. Different tissues produce different proteases so this may confer limitations on the tissues that astroviruses can infect [16,17,18]. Trypsin is produced in the highest quantity in the GI tract, and this would provide a plausible explanation as to why most human infections due to classic human astroviruses occur in that organ [18]. Nonetheless, there are two reports of classic human astrovirus serotypes causing encephalitis in humans, and in one case, the virus was detected in multiple tissues outside the GI tract, which raises the possibility that brain-derived trypsin or additional proteases could be sufficient for viral activation [19]. In contrast, some astroviruses do not require trypsin for propagation in cell culture and are known to infect tissues outside of the GI tract, including astrovirus VA1 and avian nephritis virus [20,21]. VA1 has been detected in multiple tissue types, including the GI tract, brain, serum, and from the nasopharynx, and can be propagated in primary astrocytes in cell culture without the aid of trypsin [3,22,23,24]. In total, the requirement, or lack thereof, for host proteases for the astrovirus lifecycle could play an important role in determining the spectrum of diseases a particular astrovirus genotype could cause.

## 4. Gastrointestinal Disease

The association of astroviruses with gastrointestinal disease originates from their discovery in stool samples from children with acute diarrhea [25,26]. Unique viral particles that did not fit the morphology of other known viruses were identified by electron microscopy [25,26]. Because of the starry appearance of some of the viral particles, astroviruses gained their name (astro is Latin for relating to the stars) [25,26]. Subsequently, the vast majority of astrovirus genomes have been identified from samples originating from the gastrointestinal tract of the host [1]. While this could be partially due to sampling bias, it would suggest that many astroviruses have a gastrointestinal tropism.

Transmission studies for the association between classic human astrovirus infection and gastrointestinal disease have been completed through the usage of human volunteers [27,28]. In two studies with a total of 36 volunteers, two subjects developed symptoms after ingestion of a stool filtrate containing classic human astrovirus viral particles [27,28]. In one of the studies, none of the volunteers developed symptoms after initial ingestion of a 1 mL inoculum, so the inoculum was increased to 20 mL [28]. One of the two volunteers who ingested the larger dose developed symptoms including nausea, vomiting, diarrhea, and malaise [28]. Viral particles could then be identified from the stool samples of the symptomatic volunteers [27,28]. While many volunteers did not have symptoms, many did develop an antibody response [27,28]. Interestingly, some volunteers had no symptoms and had an undetectable astrovirus antibody titer postingestion, despite their preingestion serum samples having no evidence of a preexisting antibody response, suggesting that individuals may be differentially susceptible to infection [27,28].

Other astroviruses have been associated with outbreaks of gastroenteritis in humans. An unexplained outbreak of gastroenteritis in Virginia, United States of America led to the discovery of astrovirus VA1 [22,29,30,31]. In humans, astroviruses have been quantified as being the third to fifth most common cause of viral gastroenteritis [32,33,34,35]. Human astroviruses, in general, cause a mild and self-limited gastrointestinal disease [1]. Prolonged shedding of astroviruses has been described in immunocompromised hosts, and in one case, shedding occurred over one year [30,36,37]. There are very few reports of the cellular localization of astroviruses in the context of human gastroenteritis, but in one case, the capsid of an unknown serotype of human astrovirus was localized to gut epithelial cells of the duodenum and jejunum [36]. While there has been a clear association of the classic human astroviruses with gastrointestinal disease, there is not the same association for some of the recently identified novel human astroviruses, with some studies showing an association while others not [38,39,40]. These results are striking because most adults are seropositive for both the classic and novel human astroviruses [41,42,43,44,45]. These findings would suggest the possibility of a significant number of asymptomatic infections. Alternatively, astroviruses could be the causative agent of diseases outside of the GI tract, as discussed later.

For nonhuman astroviruses, experimental data demonstrating the capacity of astroviruses causing gastrointestinal disease in other vertebrates was initially demonstrated in lambs [46], turkeys [47], and kittens [48]. Astroviruses have been subsequently identified from the GI tract of many other birds and mammals, including marine mammals [13,49,50]. However, this tissue tropism has led to a self-fulfilling prophecy. Because the gastrointestinal tract was presumed to be the primary site of astrovirus replication, previous research focused efforts on studying stool samples for discovery of novel astrovirus genomes, leading to an underappreciation of the extent to which astroviruses cause disease outside of the GI tract.

Currently, the best model organism for studying the pathogenesis of gastrointestinal disease due to astrovirus infection is the turkey poult model [51]. Inoculation of turkey poults with a turkey astrovirus causes diarrhea, allowing for the study of the cellular tropisms and pathogenesis of disease [51]. This has led to several interesting findings including the capacity of the astrovirus capsid to induce diarrhea in absence of viral replication or cell death [52]. Oral administration of the turkey astrovirus capsid leads to intestinal epithelial barrier disruption, suggesting the capsid could act as an enterotoxin [52].

Nonetheless, the turkey poult model has its limitations, and many reagents and genetic tools have not been developed for turkeys in order to study host–pathogen interactions. Astroviruses are known to infect mice and are frequently detected in most research-based mouse colonies [53]. However, murine astroviruses have not been clearly associated with a specific disease in mice, as uninfected mice excrete virus in their stool but do not develop diarrhea or systemic signs upon infection [53,54]. Murine astrovirus infection has been localized to goblet cells in mice leading to alterations in mucus production and subsequently, the gut microbiome, but these changes do not appear to cause a clear phenotype [55,56]. Interestingly, murine astroviruses may also play an important role in shaping immunity of the GI tract. Infection of a specific genotype of murine astrovirus leads to expression of interferon-lambda and is protective against further infection of mice against pathogens like murine norovirus [57]. Attempts to model human astrovirus infection in mice has failed, as inoculation of a classic human astrovirus serotype did not result in any identifiable disease and the virus could not be propagated [54].

In addition, many other astroviruses do not have a clear link to gastrointestinal disease. Initially described in 1978, inoculation of gnotobiotic calves with a bovine astrovirus strain did not result in GI disease [58]. Subsequent prevalence studies have demonstrated that bovine astrovirus prevalence rates by PCR can be as high as 100% in calves, but nonetheless, the presence is not associated with gastroenteritis [59,60,61,62]. In adult cattle, the prevalence is much lower, with 2.4% of adult cattle stool samples being positive by PCR [63]. Similarly, bats are frequently colonized by a diverse variety of bat astrovirus genomes. Up to 93% of bats harbor at least one astrovirus strain, but no clear association with disease has been made [64,65]. Some genotypes of porcine astroviruses can be present in up to 95% of fecal swabs by three weeks of age, but these piglets were otherwise healthy [66]. In surveillance of feline stool samples, 18–29% were positive for feline astrovirus, demonstrating frequent infection [67,68,69]. However, the results have been mixed for an epidemiological association between feline astrovirus and diarrhea, despite experimental evidence of the capacity of these viruses to cause diarrhea [69,70].

In summary, these results demonstrate that some astroviruses are pathogens of the GI tract and can lead to disease. However, other astroviruses are frequently detected from the GI tract of otherwise healthy vertebrates. The GI tract may be an important site of asymptomatic infection and transmission and could be an important launching pad for the dissemination of the virus to other organs, leading to subsequent diseases.

## 5. Liver Disease

The first disease association of astroviruses outside of the gastrointestinal tract was the identification of a duck astrovirus associated with hepatitis. An infectious agent causing hepatitis in ducks was described in 1965, and further testing revealed it was an astrovirus strain [71,72,73,74]. The mortality rate associated with duck hepatitis is highest in young ducklings, nearing 50% in 6–14-day-old ducklings [73,74]. In 4–6-week-old ducklings, the mortality rate drops to 10–25% [73]. Many of the ducklings died in rapid fashion, often occurring within 1–2 h of onset of signs, which can include opisthotonos [73]. Histological examination of the livers identified the widespread necrosis of hepatocytes [73]. Inoculation of healthy ducklings with liver homogenate caused hepatitis and death, providing experimental evidence for duck astroviruses causing hepatitis [73].

While the connection between duck astrovirus and fatal hepatitis in birds is strong, there is preliminary data suggesting a connection between human astrovirus infection and hepatitis. Gonzales-Gustavson et al. performed next-generation sequencing of pooled serum samples from patients with acute hepatitis [75]. Reads to astrovirus VA3 was identified in 3 out of 4 sample pools, but not in any of the control, nonhepatitis sample pools [75]. While not definitive, this is the first epidemiological link between astrovirus infection and hepatitis in humans. Further evidence suggesting a broader tropism of astroviruses to the liver is the recent identification of novel astroviruses from the livers of fish, reptiles, and amphibians [5]. In some cases, the liver was the exclusive site for detection of the novel astrovirus genotype [5]. Furthermore, in both the turkey and murine astrovirus models, astrovirus RNA can be detected in the liver [51,53]. In mice, the adaptive immune system may be important in controlling the dissemination of astrovirus infection, as Rag1 knockout mice had detectable murine astrovirus RNA from the liver, while wild-type mice had undetectable levels of RNA [53]. These results suggest that astroviruses could have the capacity to infect the liver, and that the adaptive immune response is an important component for controlling infection. Nonetheless, this area remains poorly understood in the context of humans and other mammals.

## 6. Kidney Disease

Avian nephritis virus was originally described in 1979 and was initially presumed to be a novel picornavirus [76]. Subsequent sequencing of the genome revealed that the causative agent was misclassified and was actually an astrovirus [21]. Avian nephritis virus is widely disseminated in commercial bird populations with many flocks having seropositivity rates of 100% [77,78]. Experimental infection of the virus leads to nephrosis with disease of the proximal tubules and subsequent development of interstitial nephritis [79]. Later in the disease, visceral urate deposits can be identified [79]. Other avian astroviruses have been associated with kidney diseases leading to urate crystal deposition and gout, including recent attention to outbreaks in geese from China [80,81]. This has led to considerable economic impact with infection rates nearing 80% and mortality rates around 50% [81]. Infection of goslings leads to disseminated infection with detection of virus from most tissues including the liver, heart, and brain, and these infections were associated with pathology of the kidneys and urate crystal depositions in many tissues [82]. Currently no treatment or vaccine exists for avian astroviruses.

In other mammals, no clear association has been made between astroviruses and kidney disease, but astrovirus MLB2 was detected from a urine specimen of a patient suffering from encephalitis [83]. Murine astrovirus has also been detected from the kidneys of Rag1 knockout mice [53].

## 7. Central Nervous System Disease

The identification of an astrovirus strain in brain tissue from a human patient with encephalitis in 2010 opened a new frontier on the spectrum of astrovirus-associated diseases, leading to reinvigorated interest in the pathogenicity of these viruses [84]. Subsequently, astroviruses have been associated with neurological diseases in other mammals including cattle, pigs, mink, muskox, sheep, and alpaca [3,85,86]. Currently, the prevalence of astrovirus-associated neurological diseases remains unknown.

In humans, neurological conditions like encephalitis remain diagnostic conundrums as over 60% of cases are without an identified etiology [87]. After the identification of the index astrovirus encephalitis case in 2010, a subsequent 10 cases have been published, with the most frequently identified strains being related to astrovirus VA1 and other cases caused by classic human astrovirus 1 and 4, astrovirus MLB1, and MLB2 (Table 1) [19,83,84,88,89,90,91,92,93,94]. Obtaining a brain biopsy and performing unbiased next-generation sequencing to identify potential pathogens was central to the diagnosis of many of these cases [3]. In all of the cases in which a biopsy was performed, all had histological abnormalities including neuronal apoptosis, neuronophagia, neuronal karyorrhexis, inflammatory infiltrates, or gliosis [19,84,88,89,90,91]. These cases occurred in both children and adults, with most patients having an underlying immunocompromising condition including X-linked agammaglobulinemia or hematopoietic stem cell transplant [3]. A minority of cases has occurred in otherwise healthy individuals, and it is unclear what factors led to infection in those cases [83,93,94]. So far, the case fatality rate for astrovirus encephalitis in humans is over 50%. No treatment has been approved by medical regulatory agencies for astroviruses, but in vitro data suggest that interferon, ribavirin, favipiravir, and nitazoxanide have antiviral properties, depending on the viral strain that was tested [20,95,96,97,98,99,100,101]. In a report of survival, a human patient was diagnosed with astrovirus encephalitis and subsequently treated with ribavirin, intravenous immunoglobulin, and interferon, but it is unclear what benefit these drugs had in the positive outcome [90].

The first barrier to understanding the pathogenesis of astrovirus encephalitis was the lack of cell-culture models for propagation of the recently discovered astrovirus genotypes that have been associated with encephalitis. Subsequently, astrovirus VA1, MLB1, and MLB2 have been propagated in cell culture, including in intestinal enteroids [20,98,99]. From the case reports of human VA1 encephalitis, astrovirus nucleic acid or protein has been colocalized to astrocytes and cells that have similar morphological appearance as neurons [84,88,89]. Experimental data with VA1 have demonstrated that primary human astrocytes, but not primary human neurons, support the full lifecycle of VA1 in cell culture [24]. In contrast, classic human astrovirus 4 can only cause abortive infection in primary human astrocytes, suggesting a differential capacity of astroviruses to cause central nervous system (CNS) infection [24]. The observed tropism of VA1 to astrocytes, but not neurons, in cell culture raises questions of how infection can lead to the neuronal injuries that were present in the brain biopsies. One potential mechanism is that VA1 infection leads to expression of CXCL10 from astrocytes, an important inflammatory cytokine induced by other neurotropic viruses that leads to the infiltration of immune cells and induction of neuronal apoptosis [24,102,103]. Alternatively, the primary neuronal cell culture model may not fully recapitulate neuronal cell types and conditions in vivo that are necessary for astrovirus replication, and further optimization of the conditions are needed.

Immediately after the first case report of astrovirus associated encephalitis was published, mink astrovirus was identified to be the causative pathogen in shaking mink syndrome, and the disease could be replicated by injection of infected brain material into healthy minks [104,105]. Infected minks develop a staggering gait, shaking, and ataxia [105]. These experimental findings were important to demonstrating that astroviruses are bona fide pathogens of the CNS and are not just innocent bystanders.

While the prevalence of astrovirus-associated CNS infections in humans remains understudied, there is strong evidence that bovine astroviruses are a significant cause of neurological disease in cattle. Despite the discovery of cattle astroviruses several decades ago, the association between bovine astroviruses and encephalitis was only made in 2013 [106]. Subsequently, a multitude of case reports have been published [107,108,109,110,111,112,113,114,115,116,117,118,119]. Cross-species transmission has also been identified, as a bovine astrovirus was detected from brain tissue from a sheep suffering from encephalitis [120]. In case descriptions of the afflicted cattle, many had nonspecific signs of anorexia and decreased activity, while others had overt neurological signs including tremor, dysphagia, compulsive walking, coordination deficits, and aggressive behavior [114]. These signs could evolve quickly over one day or were more prolonged, with some being observed over three weeks [114]. To better understand the larger prevalence of bovine astrovirus encephalitis, Selimovic-Hamza et al. analyzed a retrospective cohort of cattle brain tissue obtained from cases of nonsuppurative encephalitis without a previously identified etiology [113]. Surprisingly, 34% of the samples were positive for bovine astrovirus, suggesting a significant disease burden that was previously unappreciated. In a second retrospective cohort, cattle brains dating back to 1958 were also identified to be positive for bovine astrovirus, further suggesting bovine astroviruses have been afflicting cattle for the past half century [112].

Within the brain, bovine astroviruses have been detected most frequently in the brainstem but can be frequently identified in the cerebrum and cerebellum [112,113]. The hippocampus is also commonly affected, with some cases having the highest frequency of positive cells for bovine astrovirus [112,113]. For most of the bovine cases of astrovirus encephalitis, spinal cord tissue was unavailable, so it is unknown if bovine astroviruses also infect the spinal cord. At the cellular level, bovine astrovirus has been detected almost exclusively in cells that have the morphological appearance of neurons, with sporadic detection in microglial cells [112,113]. Bovine astrovirus has not been detected in astrocytes, in contrast to some of the data for astrovirus VA1 in humans and in cell culture [24,112,113]. These findings may further highlight similarities and differences in the pathogenesis of CNS infection due to different astroviruses. Neurotropic bovine astroviruses have not been isolated and cultured, preventing experimental recapitulation of the disease in cell culture and in vivo.

As the cases of bovine encephalitis were being discovered, the association of neurological diseases in pigs due to astroviruses was also established. In 2013, porcine astrovirus nucleic acid was detected in brain tissue by PCR from healthy pigs and pigs suffering from congenital tremor [121]. While there was not an association between porcine astrovirus infection and congenital tremor in that publication, this raised the question of whether porcine astroviruses were also neurotropic. Then in 2017, two simultaneous publications established the connection between porcine astroviruses and encephalomyelitis [122,123]. Boros et al. reported an outbreak of paraplegia and flaccid paralysis of young pigs from Hungary [122]. Lesions were present in the cerebellum, brainstem, and cerebellar peduncles [122]. Significant disease was observed in the spinal column, correlating with the neurological deficits of the pigs [122]. Sequencing of CNS tissue identified a strain of porcine astrovirus that was detected in multiple pigs and could be localized to neurons by in situ hybridization [122]. In the second report, Arruda et al. described pigs from the United States with hind limb weakness, quadriplegia, and convulsions in which CNS tissue was positive for a closely related porcine astrovirus [123].

Other neurological diseases have been attributed to astroviruses in sheep, muskox, and alpaca [85,86,120,124,125]. Given the frequency that astroviruses are detected from animals, there could be a greater burden of astrovirus-associated neurological diseases than what has been previously appreciated.

## 8. Respiratory Disease

To date, no clear association between astrovirus infection and respiratory diseases has been made, however, several astroviruses have been detected from nasopharyngeal (NP) swabs. All three clades of human astroviruses have been detected from human NP or oral swabs and in some cases were associated with respiratory symptoms [19,83,92,126,127,128]. Other mammals have also been positive from NP swabs including camels, pigs, and cattle [122,129,130,131,132]. In a case-control series of bovine respiratory disease (BRD), four cattle with BRD were positive for bovine astrovirus, while all 50 controls were negative [132]. While this result did not reach statistical significance and there was the confounding factor of coinfections with other respiratory viruses, this does raise the possibility of astroviruses causing respiratory diseases. Similarly in a small study in pigs suffering from unexplained respiratory disease, porcine astroviruses were detected on nasal swabs [122]. Novel astroviruses have also been detected from the gills of fish and lungs of reptiles and amphibians, providing an evolutionary link for astroviruses being present in the respiratory tract of a diverse range of vertebrates [5].

The presence of astroviruses from respiratory samples also raises the question of if the virus can be transmitted through means other than the fecal–oral route, which is assumed to be the primary route of transmission. In one of the studies of porcine-associated CNS disease, 80% of the affected pigs had detectable virus from nasal swabs, while none of their stool samples were positive [122]. In another study, 25% of pig lungs were positive for porcine astroviruses [133]. Further development of in vivo models of astrovirus infection will allow transmission studies to be conducted to better understand to what extent, if any, astroviruses can be spread through respiratory secretions and cause subsequent disease.

## 9. Systemic Disease

Chickens are prone to a disease known as runting-stunting syndrome, leading to decreased body weight [134]. Multiple viruses have been associated with this syndrome, including avian nephritis virus and chicken astrovirus [134]. Recently, a unique syndrome in chickens has been further attributed to chicken astroviruses, named “white chicks hatchery syndrome” [135]. In this disease, newly hatched chicks have white plumage with pale legs and beaks [136]. Death often occurs within a day after hatching [136]. In afflicted chickens, the liver is enlarged, the gallbladders are distended, and intestines are filled with gas and liquid [136]. Chicken astrovirus is most often isolated from the gizzard, intestines, lungs, kidneys, pancreas, spleen, and yolk, with less frequent detection from the liver, heart, brain, and thymus [136]. While the disease seems to predominate in the liver and GI tract, the primary pathogenesis of this disease is poorly understood. Experimental injection of chicken eggs with chicken astrovirus leads to viral replication and is associated with reduction in growth of the embryos [137]. Taken together, the extent by which the chicken astrovirus disseminates in the afflicted host demonstrates a very broad tissue tropism in a young avian host.

Astroviruses have been detected from the blood of human subjects. In three cases, children presented with fever, and two out of the three children had respiratory symptoms [138,139]. Testing identified astroviruses MLB1 or MLB2 from plasma specimens from the subjects [127,138]. In screening of human blood products, MLB2 was also identified from a human donor, who was reportedly asymptomatic at the time of blood donation [140]. Other human astroviruses have been detected from the serum of patients with or without neurological diseases [19,75,83,88,92,139,141]. These cases raise the question that astroviruses may cause nonspecific illnesses in humans and may be detected from the blood.

## 10. New Frontiers of Astrovirus Tissue Tropisms and Diseases

Despite the discovery of the first astrovirus over 40 years ago, astroviruses have not received the same attention as other viral RNA families. Part of this disinterest was driven by the assumption that astroviruses only cause a self-limited gastrointestinal disease in humans and other vertebrates. However, discoveries in the past decade have highlighted the unappreciated disease burden these viruses have in humans, agriculturally important livestock, and many other organisms. These findings are the proverbial “tip of the iceberg” of the extent of diseases caused by astroviruses. Implementation of unbiased next-generation sequencing will continue to be a driver of the discovery of novel astrovirus genotypes. A greater understanding of the epidemiology and disease burden associated with astrovirus infection is needed.

At this point, astroviruses have been associated with diseases of many of the major organs of vertebrates and new disease associations will likely continue to emerge. One organ system that raises further questions is the detection of astroviruses from tissues of the cardiovascular system. In a small number of studies, astrovirus RNA has been detected in infected human and animal hearts, including pigs, ducks, chickens, and geese [19,66,142,143,144]. In addition, histological findings of myocarditis or epicarditis were identified from two pigs infected with porcine astrovirus [66]. Similarly, it is unknown whether astroviruses can infect cells of the immune or hematological systems. Astroviruses have been detected by PCR from splenic tissue, and, in some cases, viral protein has been detected by immunofluorescence in specimens from the spleen [51,53,82,143]. In addition, the detection of astroviruses in stool has been associated with an increased risk of immune thrombocytopenia in children [145]. These potential tissue tropisms highlight future directions that must be further experimentally tested with in vivo models of astrovirus infection. In addition, the vast majority of astrovirus strains remain uncultured, limiting the extent at which the biology and pathogenesis of infection can be experimentally elucidated for many other astrovirus genotypes.

Taken together, astroviruses remain a relatively understudied viral family but are a field ripe for major discoveries that could have major implications for human and animal health.

## Figures and Tables

**Table 1:** Descriptions of the published cases of neuroinvasive disease in humans caused by astroviruses.

Astrovirus Genotype	Patient Age	Sex	Location	Past Medical History	Positive Assay(s)	Astrovirus Positive Specimens	Outcome	Year of Publication	Reference
VA1	8 mos	Female	UK	Hematopoietic stem cell transplant	NGS/PCR	Brain	Death	2016	[91]
VA1	18 mos	Male	UK	Hematopoietic stem cell transplant	NGS/PCR/IHC	Brain, CSF, stool, serum	Death	2015	[88]
VA1	14 yrs	Male	France	X-linked agammaglobulinemia	NGS/PCR	Brain	Recovery	2015	[90]
VA1	15 yrs	Male	USA	X-linked agammaglobulinemia	NGS/PCR/IHC	Brain	Death	2010	[84]
VA1	42 yrs	Male	UK	Hematopoietic stem cell transplant	NGS/PCR/ISH	Brain/CSF	Death	2015	[89]
MLB1	4 yrs	Male	Japan	Hematopoietic stem cell transplant	NGS/PCR	CSF, serum, stool, urine, oropharyngeal swab	Recovery	2016	[92]
MLB2	21 yrs	Female	Switzerland	Previously healthy	NGS/PCR	CSF, stool, urine, plasma	Recovery	2016	[83]
MLB2	29 yrs	Female	USA	Previously healthy	NGS	CSF	Recovery	2019	[93]
MLB2	37 yrs	Male	Switzerland	Hematopoietic stem cell transplant	PCR	CSF, stool, plasma	Death	2016	[83]
Human astrovirus serotype 1	16 mos	Female	Germany	Previously healthy	PCR	CSF	Recovery	2019	[94]
Human astrovirus serotype 4	3 mos	Male	Switzerland	Hematopoietic stem cell transplant	PCR	Brain, plasma, skin, heart, lung, spleen, bone marrow, kidney, stool, small intestine	Death	2011	[19]

## Data Availability

Data sharing not applicable.

## Supplementary Materials

The following are available online at https://www.mdpi.com/article/10.3390/v13050732/s1, Supplementary File: List of astrovirus sequences used for Figure 2.
