# Supplementary material for: Beyond the Gastrointestinal Tract: The Emerging and Diverse Tissue Tropisms of Astroviruses
Source: Viruses. 2021 Apr 22;13(5):732. doi: 10.3390/v13050732 (PMC8145421; doi:10.3390/v13050732)
Supplement: Supplementary file 1 [file viruses-13-00732-s001.zip › viruses-1173824-supplementary.pdf]

Supplement: List of 53 astrovirus protein sequences used in Figure 2

Human Astrovirus 1 (NP_059444.1), HumanAstrovirus 2 (AZB52195.1), Human Astrovirus 3 (AZB52198.1), Human Astrovirus 4 (AZB52201.1), Human Astrovirus 5 (AZB52204.1), Human Astrovirus 6 (AZB52207.1), Human Astrovirus 7 (AZB52210.1), Human Astrovirus 8 (AAF85964.1). Astrovirus VA1 (YP_003090288.1), Astrovirus VA2 (ACX83591.2), Astrovirus VA3 (YP_006905860.1), Astrovirus VA4 (YP_006905857.1), Human astrovirus BF34 (YP_009047080.1), Astrovirus MLB1 (YP_002290968.1), Astrovirus MLB2 (YP_004934010.1), Astrovirus MLB3 (YP_006905854.1), Mamastrovirus 2-feline (YP_009052462.1), Mamastrovirus 3 Porcine (YP_009094279.1), Porcine astrovirus- encephalomyelitis (ARR73572.1), Mamastrovirus 4-California sea lion 2 (YP_009380534.1), Mamastrovirus 5-Canine astrovirus (YP_009666026.1), Mamastrovirus 7-Bottle nose dolphin 1 (YP_009664776.1), Mamastrovirus 10-Mink (NP_795336.1), Mamastrovirus 11-California sea lion 1 (ACR54272.1), Mamastrovirus 12-Bat (YP_009664780.1), Mamastrovirus 13-Ovine (NP_059946.1), Mamastrovirus 13-musk ox (QDA34115.1), Mamastrovirus 13-ovine encephalitis (SIP85307.1), Mamastrovirus 14-Bat (NP_059946.1), Mamastrovirus 15-Bat (YP_009664784.1), Mamastrovirus 16-Bat (YP_009664786.1), Mamastrovirus 17-Bat (YP_009505809.1), Mamastrovirus 18-Bat (YP_009664789.1), Mamastrovirus 19-Bat (YP_009664791.1), Murine astrovirus (YP_006843892.1), Bovine-CH13 (NC_024498.1), Bovine-S1 (AGO50636.1), Bovine astrovirus BRD (AJE25858.1), Bastrovirus 7 (YP_009422197.1). AAstV: Turkey Astrovirus-poult (AF206663.2), Chicken astrovirus/avian nephritis virus 1 (ANV1; NP_620618.1), Chicken Astrovirus/Avian Nephritis virus 1 (ANV1; AB033998.1), Avian Nephritis virus 2 (ANV2; AFW05401.1), Turkey astrovirus 1 (NP_059949.1), Turkey astrovirus 2 (NP_987088.1), Duck astrovirus (YP_002728003.1). Musa acuminata plastrovirus 2 [9], Beihai astro-like virus (YP_009333213.1), Changjiang astro-like virus (YP_009336809.1), Beihai fish astrovirus 1 (AVM87487.1), Wenling righteye flounders astrovirus (AVM87608.1), Guangdong Chinese watersnakea strovirus (AVM87610.1), Hainan black-spectacled toad astrovirus 2 (AVM87165.1),
